# Interspecific differences in the behavioral response of ticks exposed to radiofrequency electromagnetic radiation

**DOI:** 10.1007/s10493-023-00847-7

**Published:** 2023-10-11

**Authors:** Miroslav Baňas, Lívia Šofranková, Juraj Kurimský, Marek Pavlík, Mário Pikalík, Viktória Majláthová, Roman Cimbala, Natália Pipová, Liliana Wurfl, Igor Majláth

**Affiliations:** 1 Institute of biology and ecology, Pavol Jozef Safarik University in Kosice, Srobarova 2, Kosice, 041 80 Slovak Republic; 2 Department of Electrical Power Engineering, Faculty of Electrical Engineering and Informatics, Technical University of Kosice, Masiarska 74, Kosice, 041 20 Slovak Republic; 3 Boston Children´s Hospital, 300 Longwood Ave, Boston, MA 02115 USA

**Keywords:** Ticks, Behavior, Preference, Electromagnetic radiation, Interspecies and intersex differences

## Abstract

Artificial electromagnetic radiation is a new environmental factor that affects animals. Experiments with the effect of radio frequency electromagnetic radiation were focused on both vertebrates and invertebrates. Ticks showed a significant affinity to radiation. Our study is a continuation of this research and its aim was to monitor the effect of radiation on the behavior of four tick species: *Ixodes ricinus*, *Dermacentor reticulatus*, *Dermacentor marginatus* and *Haemaphysalis inermis*. In total 1,200 ticks, 300 of each species, were tested in modules allowing the choice of an exposed or shielded area. During the test, the ticks were exposed to electro-magnetic radiation of 900 MHz for 24 h. The position of the individuals was recorded and we evaluated the obtained data statistically. We observed a significant preference to the exposed area in both sexes of *I. ricinus*. Males of *D. reticulatus* and *D. marginatus* also showed an affinity to radiation, but not females of both species, nor females and males of *H. inermis*. The results of the study support the assumption that ticks perceive the electromagnetic field and the observed differences in their response have the potential to help understand the mechanism of perception.

## Introduction

Radio-frequency electromagnetic radiation (RF-EMR) does not have enough energy to have an ionizing effect on molecules and atoms in the cells of living organisms (Cleveland and Ulcek 1999). Nonetheless, scientific papers focused on the impact of non-ionizing electromagnetic radiation on animals indicate possible non-thermal effects influencing memory, learning and locomotion (Narayanan et al. 2019).

Among vertebrates, birds and rodents are the most commonly researched groups (Balmori 2005; Mokarram et al. 2017; Thalau et al. 2005) with the most studied parameters being reproduction, development, behavior and population shifts. The effect of RF-EMR on invertebrates is most often surveyed in insect models, particularly honeybees, fruit flies, cockroaches and ants (Cucurachi et al. 2013). During an experiment with honeybees (*Apis mellifera* subsp. *carnica*), authors observed the influence of an active mobile phone placed in a hive on the sounds of the honeybee colony. Analysis of audio recordings revealed that after 25–40 min of exposure to 900 MHz radiation, colonies begin to emit sounds with higher frequency and amplitude, similar to the sounds of bee workers in danger or under stress, then became quiet after the end of exposure. No similar reactions were recorded in bee colonies in which the mobile phone was persistently inactive (Favre 2011). Further studies found RF-EMR caused a decline in colony strength, egg laying rate, and activity of workers on honeycombs in addition to reduction in the success of pupal development (Kumar et al. 2011; Odemer and Odemer 2019; Sharma and Kumar 2010). Ecologically focused experiments with fruit flies (*Drosophila melanogaster*) were mainly centered on reproduction success and growth. Laboratory assays with variable parameters (frequency and power density of RF-EMR or duration of exposure) found a significant decrease in reproduction rate and ovarian size in the irradiated group when compared to unexposed fruit flies (Panagopoulos 2012; Panagopoulos et al. 2004; Panagopoulos and Margaritis 2010); conversely, results of a study from Weisbrot et al. (2003) showed a positive effect on reproduction: compared to the control, the number of offspring increased in groups exposed to radiation (900/1900 MHz) emitted by mobile phones. Exposure of low frequency EMR (1.2 MHz) also disrupts the magneto reception and orientation of American cockroaches (*Periplaneta americana*) (Vácha et al. 2009) and 900 MHz radiation depresses the association of ants (*Myrmica sabuleti*) between food and olfactory as well as visual cues (Cammaerts et al. 2012).

The idea of investigating the effect of electromagnetic radiation on ticks is relatively new, therefore the number of published studies is limited. Electromagnetic radiation at microwave frequency caused a 3–20 days delay in *Hyalomma asiaticum* larval hatching and reduced the survival rate of unfed larvae and nymphs by 4–10 days (Korotkov et al. 1996). Later experiments found a relation between the effect of microwave radiation and ambient temperature. After exposure to radiation at 22 °C, a suppression in fed larvae and nymphs was observed. On the contrary, at 14 °C, radiation had a stimulating effect on larval development (Korotkov et al. 2000). A study focused on *Dermacentor reticulatus* surveyed the influence of RF-EMR (900 MHz) on larvae hatched from radiation-exposed eggs. Larvae from a group exposed for 60 min were larger in total body length, length of gnathosoma with scutum, total body width and width of basis capituli, compared with the control and groups irradiated for 30 and 90 min (Vargová et al. 2021).

The first behavioral observation of ticks under the influence of RF-EMR was conducted on *D. reticulatus*. Ticks reacted with immediate locomotor activity and previously unobserved body jerking (Vargová et al. 2017). Further experiments using an RST (radiation shielded tube) test found affinity of both *D. reticulatus* (Vargová et al. 2018) and *Ixodes ricinus* (Frątczak et al. 2020) to exposed parts of the apparatus irradiated by 900 MHz EMR. Moreover, the passed trajectory and occurrence of *I. ricinus* in a modified radiation-shielded circular open field arena were significantly longer under radiation of the same frequency (Vargová et al. 2022).

The aim of our current study is to compare the response to EMR with a frequency of 900 MHz in RST tests of males and females of four sympatrically occurring tick species. The objective is also to expand deficient understanding of tick reaction by implementing, to our knowledge, the first observation of behavior under the influence of RF-EMR in *Dermacentor marginatus* and *Haemaphysalis inermis*.

## Materials and methods

### Ticks

For the objectives of our experiment, we gathered individuals of *I. ricinus*, *D. reticulatus*, *D. marginatus* and *H. inermis*. Questing ticks were collected from vegetation using white cotton flags in eastern Slovakia. Adult ticks were sorted based on species and sex and kept in 50 ml polypropylene tubes with sufficient air humidity (> 90% RH) and a natural light regime. For every species, 150 males and 150 females were tested. Including all four species, 1200 individuals altogether were used for the purposes of our experiments.

### Test modules and anechoic chamber

**Fig. 1:**
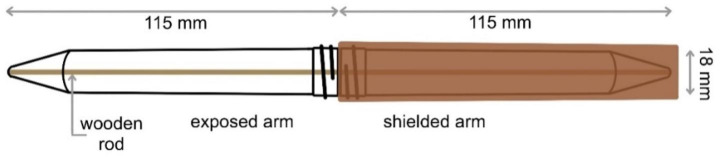
Schematic diagram of radiation shielded tube (RST) module with an exposed and a shielded portion. Shielding of the radiation is ensured by a 1-mm-thick layer of copper. A moist wooden rod was used only when testing *Ixodes ricinus* and extended through the entire module touching both ends of the tubes

Our testing apparatus, a modified radiation shielded tube (RST) originally designed by Vargová et al. (2018), was composed of two 115-mm-long polypropylene tubes with a diameter of 18 mm. Half of every module (one of the tubes) was shielded (SH) from radiation by 1-mm-thick copper cover, whereas the second portion was exposed (EXP) (Vargová et al. 2018). During *I. ricinus* tick testing, the modules were modified by adding a moist thin wooden rod extending from one end of tube to the other (Fig. 1). Rods were evenly moistened by immersion in demineralized water and quickly dried on filter paper. Modules were attached to the wooden base with two plastic holders in a horizontal position. When testing each particular species and sex, 30 RST modules were used and five ticks were placed in each module. The orientation of the modules (whether the shielded portion was on the right or on the left) was randomized.

The experiment took place in an anechoic chamber (Model 1710–100, Comtest, Zoeterwoude, The Netherlands) to exclude the possibility of ticks interacting with the surrounding artificial electromagnetic field. RF-EMF was emitted by the Double-Ridged Waveguide Horn Antenna HF907 with vertical polarization of the electric part of the RF field (Rohde and Schwarz, Munich, Germany) connected to a generator of signal (N5183A, Agilent, Malaysia). Our selected frequency of 900 MHz is widely used in mobile phone communication. The antenna as a source of radiation was placed 2 m from the stand carrying the RST modules. At the location of the RST modules, a power density of 4,244 W/m^2^ was applied. This was verified using a spectrum analyzer (Spectran HF-60,105, Aaronia, Strickscheid, Germany). The electromagnetic field intensity was 40 V/m. Ticks were exposed at 22 °C and 60% RH.

### Experimental procedure

Tested ticks were hydrated for 24 h before the experiment in Petri dishes on moist filter paper at standard laboratory temperature and natural light conditions. The test itself took place in an anechoic chamber that was in complete darkness. Five ticks of the same sex and species were placed in the middle of every RST module and exposed to RF-EMR. After 24 h, the positions of individuals were noted, i.e., whether in the exposed or shielded portion of the module at the time of assessment.

### Statistical analysis

Due to the distribution of our data, we used several statistical tests to evaluate the obtained results. To compare the number of exposed (EXP) and shielded (SH) individuals, we used Pearson’s χ^2^ test. A cross-tabulation test was used to determine whether there was a significant association between sex and reaction to irradiation of RF-EMR. In addition, we used a generalized linear model (GLM) with binomial distribution and logit input to detect interactions between independent variables (specifically species, sex, and species*sex) with the dependent variable ‘irradiation reaction’.

## Results

The ticks in our experiment reacted to the presence of RF-EMR, which is indicated by their distribution within the RST modules. We found differences in their responses and preference for the exposed or shielded part of the modules at the species level as well as between sexes. We did not observe a significant preference for the shielded arm of the RST module in any of the species and sexes. Therefore, we do not assume a repulsive effect on ticks during exposure of radiation with used parameters.

### *Ixodes ricinus*

**Fig. 2:**
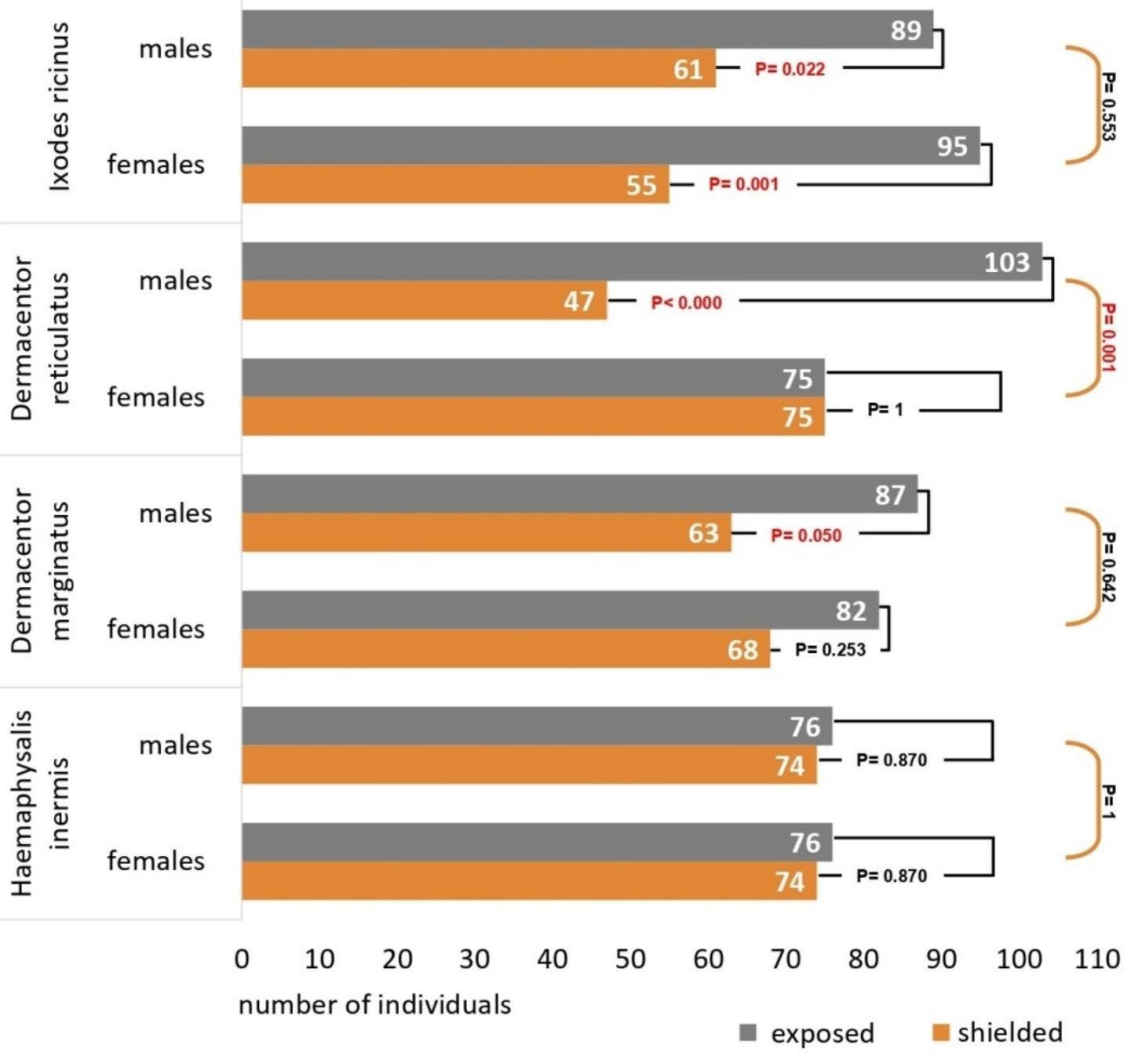
Recorded number of ticks in the exposed/shielded part of the radiation shielded tube (RST) modules after 24 h of radio-frequency electromagnetic radiation (RF-EMR) exposure for four tick species. P values from the comparison of exposed/shielded area preference (within a species and sex) were determined by χ^2^ test and are connected by a black line. Differences in the reaction of males and females (within a species) were analyzed by cross-tabulation tests and are connected by an orange line

In *I. ricinus*, we observed affinity to the exposed area (Fig. 2). Among 150 individuals of each sex, 89 males and 95 females chose the irradiated portion, vs. 61 males and 55 females preferred the shielded arm (males: χ^2^ = 5.227, df = 1, P = 0.022; females: χ^2^ = 10.667, df = 1, P = 0.001). Cross-tabulation tests showed no association between sex and reaction to the irradiation of RF-EMR (two-tailed test: P = 0.55).

### *Dermacentor reticulatus*

The *D. reticulatus* males showed the strongest affinity to the electromagnetic field in our experiment (Fig. 2). Out of 150 male ticks in the test, up to 103 (69%) chose the exposed portion, 47 preferred shielded part (χ^2^ = 20.907, df = 1, P < 0.000). Conversely, the females were evenly distributed between the exposed (75 individuals) and shielded (75 individuals) areas. The crosstab test determined that there is a significant association between sex and reaction to exposure of radiation (two-tailed test: P = 0.001).

### *Dermacentor marginatus*

A similar trend (though not as strong) in sex differences in reactions to RF-EMF was found in *D. marginatus* (Fig. 2). The exposed portion of the module was preferred by 87 males and 82 females, vs. 63 males and 68 females in the shielded area (males: χ^2^ = 3.840, df = 1, P = 0.050; females: χ^2^ = 1.307, df = 1, P = 0.25). Cross-tabulation tests did not find an association between sex and the reaction to radiation (two-tailed test: P = 0.64).

### *Haemaphysalis inermis*

We did not observe any response or preference of the EXP/SH area during exposure to radiation in *H. inermis* ticks (Fig. 2). Equal numbers of males and females chose the exposed area vs. the shielded part (76 vs. 74 for both males and females; both χ^2^ = 0.27, df = 1, P = 0.87).

### Interspecies differences

A generalized linear model with binominal distribution was used for the determination of significant differences between the dependent variable, irradiation, and three independent variables: species, sex, and species*sex. An omnibus test approved the use of the GLM test (χ^2^ = 19.657, df = 7, P = 0.006). Two variables were significant: species (χ^2^ = 8.089, df = 3, P = 0.044) and species*sex (χ^2^ = 9.143, df = 3, P = 0.027). Cross-tabulation tests on all species together found significant differences between position choice (EXP vs. SH) when considering males and females as one group (P = 0.048), and when considering males separately (P = 0.017), but not when considering females separately (P = 0.077).

## Discussion

Relative air humidity, light, and temperature are known factors affecting the locomotor activity of ticks (Alekseev et al. 2000; Lees 1948). RF-EMR is considered to be another possible factor that affects the behavior and movement of ticks (Vargová et al. 2017). The results of our experiment show that there are differences in the reaction of ticks to 900 MHz of artificial electromagnetic radiation, with regard to species and (in some cases) sex. Tested *I. ricinus* had a distinct preference for the unshielded area. This finding coincides with results from an earlier study from Frątczak et al. (2020) on the same species: males and females were attracted to the irradiated area, and the attractive effect of radiation was stronger for ticks infected with *Rickettsia* spp.

The first similar behavioral study on *D. reticulatus* was carried out with the irradiation of ticks on a glass rod. In the presence of RF-EMR, significantly higher locomotor activity was observed. Ticks reacted with specific whole-body jerking which was performed more frequently in females; similarly, ticks reacted with a jerking of the first pair of legs which was observed more frequently in males (Vargová et al. 2017). Results of further study using RST modules indicated an escape reaction to the shielded arm of the module when emitting radiation with a frequency of 5000 MHz and sex-independent preference for the unshielded area at 900 MHz of radiation (Vargová et al. 2018). In contrast, the data from our experiment indicate a significant sex-associated response, with a high affinity for radiation in males and no apparent preference in females. The cause of the disparity between our current findings and the conclusions of a previous experiment remains presently unclear. A possible explanation is the variation in the parameters of the radiation applied during the experiments. Laboratory tests on ticks from the literature that applied 900 MHz worked with a significantly lower intensity (0.6 V/m). The intensity of the radiation we used was 40 V/m.

Interestingly, males and females of *H. inermis* showed no significant reaction to RFEMR. To explain the non-response to radiation, further observations are necessary to rule out the possibility that the ticks are insensitive only to the radiation with the parameters we used. Based on the current limited knowledge of tick behavior under the influence of electromagnetic radiation, we cannot decide which radiation parameters (frequency, power density, magnetic field intensity) or their interaction have an attractive vs. repulsive effect on ticks. Cucurachi et al. (2013) compared the applied power density in studies devoted to the effect of radiation on insects and revealed no clear doseresponse association. Both low and high dosages produced significant effects (Cucurachi et al. 2013). If the response to irradiation was truly absent in *H. inermis*, its comparison with species that do react could contribute to clarifying the tick perception of RF-EMR.

Experiments with ticks of different species under the influence of radiation indicate that in most cases ticks react and their responses are different. Further potential research in this area should move from asking if they react at all to questions of why this ability could be beneficial for them and how ticks perceive radiation. All living organisms produce their own weak endogenous electromagnetic field with a specific frequency (Elizarov 1997; Liboff 2004), thus the perception of RF-EMR may be related to host-seeking activity. The possible use of ‘electromagnetic sense’ may lead to faster detection of hosts than the use of smell (Vargová et al. 2017).
